# Supplementary material for: Large-scale outbreak of Chikungunya virus infection in Thailand, 2018–2019
Source: PLoS One. 2021 Mar 10;16(3):e0247314. doi: 10.1371/journal.pone.0247314 (PMC7946318; doi:10.1371/journal.pone.0247314)
Supplement: S1 File — (DOCX) [file pone.0247314.s001.docx]

**S1 File: GenBank accession numbers**

The partial E1 gene sequences of the CHIKV isolates in Thailand during the October 2018 to February 2020 outbreak are available in GenBank (accession numbers: MT346067, MT346068, MT348938-MT348940, MT348943, MT348945-MT348947, MT348952-MT348954, MT348958, MT348961-MT348973, MT348975-MT348977, MT348979-MT348987, MT348989-MT348995, MT348999-MT349002, MT349004-MT349010, MT349015-MT349017, MT349020, MT349021, MT349024, MT349027, MT349029-MT349031, MT349033, MT349035-MT349039, MT349041-MT349045, MT349047-MT349049, MT349052-MT349059, MT349061-MT349063, MT349066, MT349069-MT349073, MT349076, MT349078, MT349079, MT349081-MT349086, MT349091, MT349092, MT349094-MT349098, MT349100, MT349102, MT349104-MT349106, MT349109-MT349115, MT349119, MT349125, MT349128, MT349130, MT349132-MT349134, MT349136-MT349140, MT349143, MT349144, MT349146, MT349148, MT349149, MT349151-MT349156, MT349159-MT349161, MT349163-MT349166, MT349168-MT349171, MT349173-MT349184, MT349186-MT349190, MT349192-MT349196, MT349199, MT349200, MT349202, MT349203, MT349205-MT349215, MT349217-MT349224, MT349226-MT349228, MT349230-MT349236, MT349238, MT349240-MT349244, MT349246, MT349247, MT349249, MT349251-MT349253, MT349255-MT349259, MT349261, MT349263-MT349276MT349278-MT349281, MT349284, MT349286, MT349288, MT349289, MT349291). The whole genome sequences of CHIKV were deposited in GenBank (accession numbers: MT495605-MT495608, MT640255, and MT640256).
