# Supplementary material for: Large-scale outbreak of Chikungunya virus infection in Thailand, 2018–2019
Source: PLoS One. 2021 Mar 10;16(3):e0247314. doi: 10.1371/journal.pone.0247314 (PMC7946318; doi:10.1371/journal.pone.0247314)
Supplement: S2 File — (PDF) [file pone.0247314.s002.pdf]

**S2 File: Positive cases detected by real-time PCR and rapid IgM/IgG and ELISA IgM/IgG according to the days after onset**

Day(s) after disease onset

| Positive (%) | 1 | 2 | 3 | 4 | 5 | 6 | 7 | 8 | 9 | 10 | 11 | 12 | ≥ 13 | Total |
|---|---|---|---|---|---|---|---|---|---|---|---|---|---|---|
| PCR | 87.5 | 94.5 | 94.9 | 93.3 | 86.4 | 42.9 | 37.5 | 37.5 | 30.0 | 0.0 | 25.0 | 16.7 | 2.9 | 71.3 |
| Rapid IgM | 18.8 | 6.8 | 6.8 | 20.0 | 45.5 | 78.6 | 100.0 | 75.0 | 90.0 | 100.0 | 100.0 | 100.0 | 97.1 | 38.4 |
| Rapid IgG | 6.3 | 2.7 | 3.4 | 6.7 | 4.5 | 0.0 | 0.0 | 25.0 | 20.0 | 33.3 | 25.0 | 50.0 | 64.7 | 12.9 |
| ELISA IgM | 12.5 | 5.5 | 5.1 | 20.0 | 50.0 | 78.6 | 100.0 | 75.0 | 90.0 | 100.0 | 100.0 | 100.0 | 100.0 | 38.1 |
| ELISA IgG | 6.3 | 4.1 | 0.0 | 4.4 | 4.5 | 0.0 | 0.0 | 37.5 | 50.0 | 33.3 | 75.0 | 100.0 | 91.2 | 18.1 |

| No. of positive samples | 1 | 2 | 3 | 4 | 5 | 6 | 7 | 8 | 9 | 10 | 11 | 12 | ≥ 13 | Total |
|---|---|---|---|---|---|---|---|---|---|---|---|---|---|---|
| PCR | 14 | 69 | 56 | 42 | 19 | 6 | 6 | 3 | 3 | 0 | 1 | 1 | 1 | 221 |
| Rapid IgM | 3 | 5 | 4 | 9 | 10 | 11 | 16 | 6 | 9 | 3 | 4 | 6 | 33 | 119 |
| Rapid IgG | 1 | 2 | 2 | 3 | 1 | 0 | 0 | 2 | 2 | 1 | 1 | 3 | 22 | 40 |
| ELISA IgM | 2 | 4 | 3 | 9 | 11 | 11 | 16 | 6 | 9 | 3 | 4 | 6 | 34 | 118 |
| ELISA IgG | 1 | 3 | 0 | 2 | 1 | 0 | 0 | 3 | 5 | 1 | 3 | 6 | 31 | 56 |
| No. of tests | 16 | 73 | 59 | 45 | 22 | 14 | 16 | 8 | 10 | 3 | 4 | 6 | 34 | 310 |
