# Supplementary figures and images for: Large-scale outbreak of Chikungunya virus infection in Thailand, 2018–2019
Source: PLoS One. 2021 Mar 10;16(3):e0247314. doi: 10.1371/journal.pone.0247314 (PMC7946318; doi:10.1371/journal.pone.0247314)

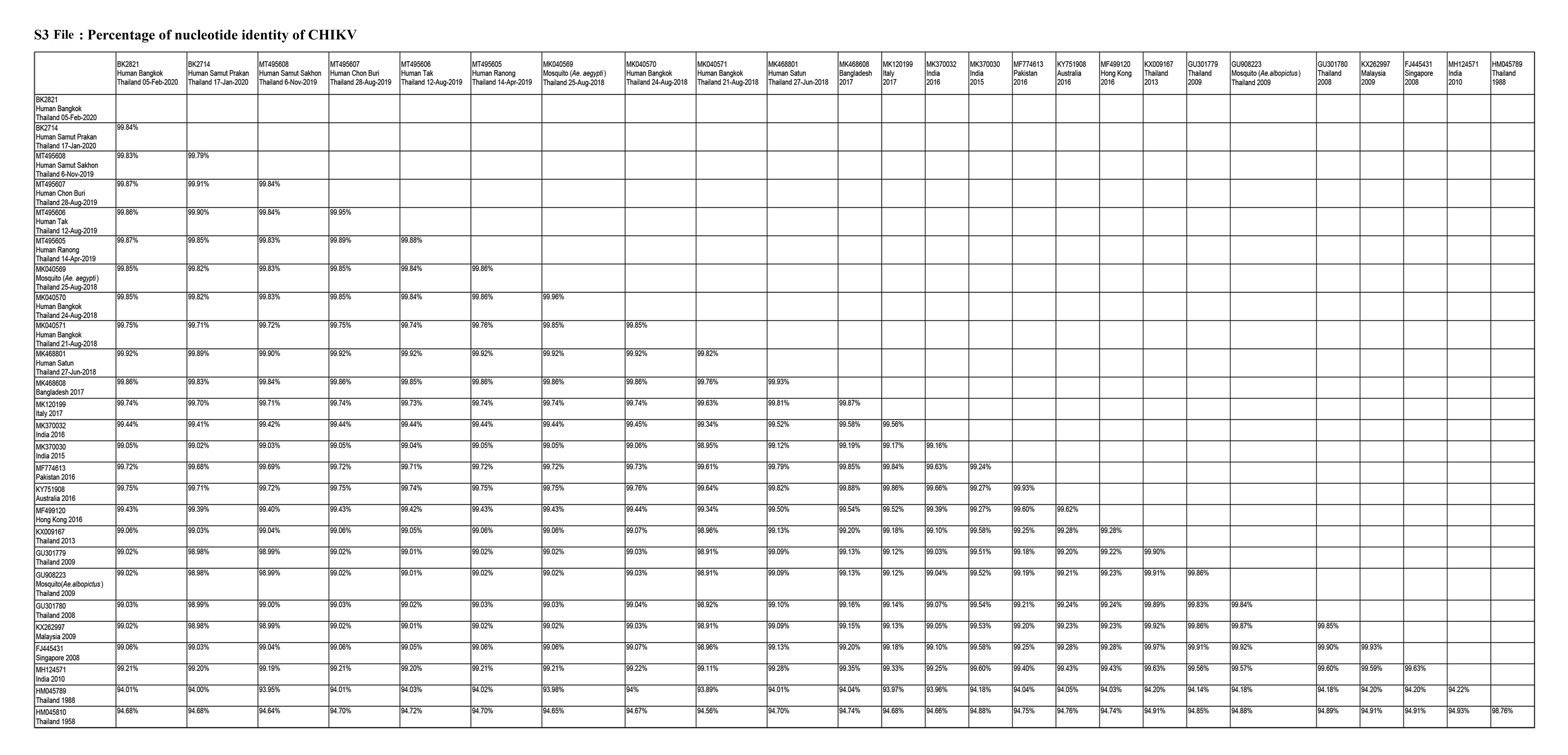

Supplement: S3 File — (TIF) [file pone.0247314.s003.tif]
