# Supplementary material for: Large-scale outbreak of Chikungunya virus infection in Thailand, 2018–2019
Source: PLoS One. 2021 Mar 10;16(3):e0247314. doi: 10.1371/journal.pone.0247314 (PMC7946318; doi:10.1371/journal.pone.0247314)
Supplement: S4 File — (DOCX) [file pone.0247314.s004.docx]

**S4 File: Amino acid changes in CHIKV Thailand strain in the 2018–2020 outbreak compared to the Thailand strain of 2008–2013**

| **Genomic region** | **Amino acid differences (%)** | **Change of amino acid(s)** |
| --- | --- | --- |
| nsP1 | 0/535 (0) | - |
| nsP2 | 5/798 (0.7) | H130Y, E145D, N495S, S539L, V793A |
| nsP3 | 1/530 (0.2) | D372E |
| nsP4 | 2/611 (0.3) | S55N, R85G |
| C | 1/261 (0.4) | K73R |
| E3 | 0/64 (0) | - |
| E2 | 3/423 (0.7) | G205S, Q252K, V264A |
| 6K | 0/61 (0) | - |
| E1 | 3/440 (0.7) | K211E, V226A, I317V |
| Total | 15/3723 (0.4) | - |

The differences in amino acids were analyzed following sequencing alignment of the Thailand CHIKV strains used in this study including Thailand virus strains of 2018-2020 (MT495605-MT495608, MT640255, MT640256, MK040569-MK040571, MK468801) by comparing with Thailand CHIKV strains of the 2008 (GU301780), 2009 (GU301779, GU908223), and 2013 (KX009167) outbreaks.
