# Supplementary material for: Large-scale outbreak of Chikungunya virus infection in Thailand, 2018–2019
Source: PLoS One. 2021 Mar 10;16(3):e0247314. doi: 10.1371/journal.pone.0247314 (PMC7946318; doi:10.1371/journal.pone.0247314)
Supplement: Attachment — Submitted filename: Response to Reviewers.doc [file pone.0247314.s006.doc]

**Review Comments to the Author**
Please use the space provided to explain your answers to the questions above. You may also include additional comments for the author, including concerns about dual publication, research ethics, or publication ethics. (Please upload your review as an attachment if it exceeds 20,000 characters)

**Reviewer #1**: (No Response)

**Reviewer #2**: The study was comprehensive, well conducted and with thorough genomic analysis that highlights the massive outbreak in 2018-20 in Thailand due to imported strain from South Asia with mutations in E1 and E2 proteins, likely associated with vector adaptation which may facilitate more efficient CHIKV transmission. The authors have made significant revisions as advised by the reviewer.

My additional comments would be:

1.The manuscript can be much improved by removing redundancies in the text.

As a suggestion, the redundancies in the text were removed as shown in the file of the revised manuscript with track changes.

2. Suggest to include vaccines as well in Lines 387, 408.

Thank you very much for the suggestion.

We included vaccines in the sentence below:

“Although CHIKV was identified in 1952, there is still no specific antiviral drug or **vaccine** against CHIKV.”

“Besides the laboratory diagnosis, the continuous surveys and studies of genetic variation of CHIKV are crucial for infection control and understanding outbreaks for further development of the **vaccine**, specific antiviral drugs or prevention of future CHIKV outbreaks.”

3. Line 57 – the commercial immunoassay is the rapid test, suggest to mention as commercial immunoassay (rapid test).

As the reviewer suggested, the word “commercial immunoassay” in the abstract was changed to commercial immunoassay (rapid test) as showed below:

“A total of 1,806 sera samples from suspected cases of chikungunya were collected from 13 provinces in Thailand, and samples were tested for the presence of CHIKV RNA, IgG, and IgM using real-time PCR, enzyme-linked immunoassay (ELISA), **commercial immunoassay (rapid test**).”

4. Line 519 – suggest to change to “IgM assay either rapid test or ELISA” would be more sensitive…….

As the reviewer suggested, the word “rapid test IgM” in the conclusions was changed IgM assay as showed below:

“whereas **IgM assay** would be more sensitive for samples obtained after six days of fever onset.”

**Reviewer #3**: The article "Large-scale outbreak of Chikungunya virus infection in Thailand, 2018–2019" presents epidemiological surveillance data for the chikungunya virus in Thailand over two years, using a large number of serum samples from patients with clinical symptoms compatible with this arbovirus. In addition to the molecular and serological diagnosis, the authors performed genomic surveillance and molecular characterization of possible mutations that led to changes in amino acids. The article was previously reviewed by other peer reviewers and the authors have already answered previous questions, as well as made the requested adjustments.

In view of what I read after the previous reviews, I consider the article to be very important, but I have some comments and questions.

Discussion

Lines 358 to 360: At the end of the first paragraph of the discussion, the authors write "We concluded that for the diagnosis of chikungunya, qualitative real-time RT-PCR was highly sensitive between days 1-5 after the onset date, and IgM testing was highly sensitive after day 7 ". This information, although it was verified during the research carried out by the authors, is not new. I request that the authors include that this information corroborates previous findings, even with the manual of the world health organization for the diagnosis of chikungunya.

Thank you very much for your suggestion.

We included the request information as the sentence below:

“These findings corroborate with previous studies and World Health Organization (WHO) guidelines for laboratory diagnosis of chikungunya.”

**Questions:**

Throughout the results and discussion, the authors describe that the evolutionary analyzes were performed by maximum likelihood (ML), using the Mega X program and some available sequences from GenBank for the creation of the reference dataset.

- Why did you choose to use Mega X for an ML analysis knowing that it has limitations and demands a long time for the construction of the phylogenetic tree? Other programs and websites are available for building ML trees in a much faster and more robust way.

We choose to use MEGA because it was performed to conduct evolutionary analysis in many studies in diverse biological fields. Moreover, this program easy to use with multiple features, including aligning sequence, test for selection model, building the phylogenetic tree, and estimating evolutionary distances.

- What was the criterion for building the dataset with a reference sample? There are many more strings available on GenBank than are used to build the tree. Has any previous analysis been carried out with representative sequences from other regions that have had major epidemics in recent years? Why didn't they include it in this analysis?

We would like to confirm the ECSA-IOL.

Most of the sequences that we selected belong to ECSA-IOL, responsible for the massive outbreak since 2006 in Asia and the virus's present strain that cause the recent outbreak in South and Southeast Asia. There is a very high probability that the virus circulating in South Asia and Southeast Asia is responsible for the current epidemic in Thailand than the virus in the other region such as Kenya (2016-2018), Republic of the Congo (2019). Moreover, the reference samples were also selected from the phylogenetic tree previously reported. Therefore, we know the cluster of reference samples used to build the dataset.

- The phylogenetic tree figure shows the three main genotypes (ECSA, West African and Asia). In the two analyzes, both with the six sequences of the complete genome and with the 251 sequences of the E1 gene, the sequenced samples grouped with other sequences of the ECSA genotype, however in the Indian Ocean (IOL) lineage. One of the IOL markers is the E1-A226V mutation, which was not found in this study. Are the authors sure that their sequences belong to IOL, since other recent sequences of the ECSA genotype from other regions, in addition to the IOL lineage, were not included in the phylogenetic reconstruction?

In this study, CHIKV belongs to ECSA-IOL lineage in phylogenetic reconstruction, including CHIKV harboring E1:226V (Singapore strain of 2008, Thailand of 2008, 2009 and 2013, Malaysia 2008 and 2009) and CHIKV possess two novel mutations in the background of E1:226A such as India 2016, Pakistan 2016, Bangladesh 2017 and Italy 2017). Our study showed that the present Thai strains were most closely related to the South Asian strain reported in 2016 and 2017 (India 2016, Pakistan 2016, and Bangladesh 2017) and Italy 2017, previously classified as ECSA-IOL. Therefore, we sure that the present Thai strains belong to the ECSA-IOL lineage. Additionally, other studies also confirmed that recent South Asian strain and Thai strain possess two novel mutations in the background of E1-226A, belong to the ECSA-IOL lineage (Intayot, Phumee et al. 2019, Lindh, Argentini et al. 2019, Eyase, Langat et al. 2020, Pyke, McMahon et al. 2020).

- Why only sequenced E1 of other samples (in addition to six complete), since a focus of the study was the amino acid substitutions of E1 and E2 proteins?

Because the previous studies showed that adaptive mutations in E1 envelope glycoproteins of CHIKV provide a fitness advantage in mosquito vector adaptation since the massive outbreak occurs in La Reunion. Moreover, our phylogenetic analysis showed a similar cluster of viral strains between the whole-genome and E1 gene. However, for future CHIKV surveillance, we plan to identify the sequence of both E1 and E2 as recent studies showed that dual mutations of E1 and E2 play a crucial role in vector adaptation.

- I suggest that in the future the authors do the phylogeographic and temporal reconstruction with a larger number of global sequences in order to demonstrate the place responsible for the introduction and to monitor and predict the route of dispersion in space and time of the strains circulating in Thailand.

Thank you for your kind suggestion.
